# Annotations

**Published:** 1892-05-07

**Authors:** 


### Annotations.

**Horse Civilisation in Shetland and Wales.**

An agricultural writer in the *Daily Chronicle* contrasts the manners of the Shetland ponies with those of the Welsh; and students who are familiar with Shetland and Wales and their peoples will find no difficulty in suggesting reasons for a certain alleged diversity. The Shetland ponies, which are so small that any man of full vigour can lift one of them from the ground, have established an admirable record for strength and character. They are said to be, like the Shetlanders themselves, of mild manners, and singular aptitude and adaptability. For circus purposes no other horses are equal to them in intelligence and docility; for children and young people they are gentle, tractable, surefo oted and trustworthy. They are equally willing to be ridden or driven; they are clever and courageous; they are never tricky, unless badly taught; and they are without rival as pets and out-door nursery companions. So much for the "Sheltie." The writer affirms that the Welsh ponies, on the other hand, are fiery and nervous, difficult to break, and that many of them become spoiled and vicious. Thus "Taffy." The reason given for this diversity in equine morals is the diversity in the manners of the human possessors of the animals. The Shetlander is mild, intelligent, rational, and reposeful in character; the Welshman is fiery, impulsive, turbulent, and defiant. Many generations of life in Shetland, and of association with its people, have given us the perfect Shetland pony. On the other hand, ages of development among the Welsh people have produced the much inferior animal of Wales. These facts, if they be facts, and the present writer can confirm them to a certain extent from his own experience, have both an amusing and a serious side. It is funny to think that the Welshman's pony is a typical Welshman in character, exactly as the Irishman's hereditary pig is said to squeak with an unmistakable brogue. There is something in it. There is an object lesson in development and the influence of environment. Facts like these are worthy of everybody's consideration; but more especially of the sober thought of the Irishman and the Welshman.

**The Return of the Sea Serpent.**

This year the sea-serpent has been born out of due time. He belongs to the sere and yellow leaf, to the season of mist and mellow fruitfulness, to the days of grouse-shooting and big gooseberries, to the time, in fact, when news is rare. Then is he yearly seen by nomad tourists on the watch for wonders. In due course come explanations of him. Letters in the newspapers follow thick and fast. "Alpha" says he isn't a serpent, but a fish, and is learned if irrelevant on the subject of conger eels. "Beta" mocks the eel hypothesis, claims the serpent as a cetacean, and goes off to seals and Behring's Straits. "Gamma" laughs at both, and disparages the serpent as a mere trail of sea-weed; and "Delta" declares that he is only a chain of distant rocks seen through a haze. He has seen a procession of porpoises, a lone surviving plesiosaurus, a gigantic octopus —anything, in short, but a real sea-serpent. But this year the sea-serpent may proudly raise his head and flap his tail; after centuries of scepticism and misrepresentation he has made a convert at last. Dr. A. C. Oudemans, of the Zoological Society of the Netherlands has espoused his cause. He has collected and collated every mention made of him in ancient and modern times. Aristotle, Pliny, and Aulus Gellius; Walter Scott, Philip Henry Gosse, and Andrew Wilson, besides scores of others, are laid under contribution to decide whether he is a reality or a myth. Dr. Oudemans has written a book, and written it in English too, in which he overhauls all the literature on the subject, tells the attempts made to discredit the sea-serpent, and the various explanations given by different naturalists. He also exposes the false claims made by the "animal of Strousa" and others to be sea-serpents. For it is not to every large, long, floating phenomenon that Dr. Oudemans will give the time-honoured name. His sea-serpent, so far as we can make out from his prospectus, is not of the common fraudulent kind, but has been evolved entirely out of his own inner consciousness. It is the child of his imagination, and therefore he can rightly dilate, in his last chapter, with parental fondness on its "taking notice," or its "not taking notice," like other babies; its timidity and its fearlessness, its "playsomeness" and its fury. He knows it down to the ground, or down to the bottom of the sea; and there is no doubt that anyone who will pay "£1 5s." for his account of it, will obtain for his money something unique in biological literature.

**Thankless Unpopularity.**

Now that the Royal Commission has dealt a serious blow at compulsory vaccination by deciding against the continuance of cumulative penalties, it is very desirable that the members of the medical profession should look at the whole question from standpoints other than medical. It is honourable to incur odium in a cause which is both just and wise, but it is foolish to run one's head against stone walls unless there is reasonable justification for it. Vaccination in itself is a purely medical question; the compelling of men and women to submit their children for vaccination has nothing to do with medicine; it is a State question; a question of public policy pure and simple. As a measure of prophylaxis which is universally applicable, and which would be universally useful if universally applied, medical men, holding the faith they do, must stand loyally by vaccination. They must prove the sincerity of their convictions by submitting themselves and their children to the operation, and by teaching and persuading all whom they can influence to accept its benefits. This is legitimate; it is rational; it commends itself to candid minds; it influences favourably even uncandid and prejudiced minds; it takes the wind out of the sails of badly instructed agitators. Canning, whose genius for statesmanship will be admitted, recognised the fundamental difference between vaccination as a prophylactic voluntarily accepted, and vaccination forced upon an unwilling public at the point of the policeman's baton. Said Canning: "Although I consider the discovery of vaccination to be of the very greatest importance, yet I cannot imagine any circumstances whatever that would induce me to follow up the most favourable report of its infallibility with any measure for its compulsory infliction." Mr. John Bright, half a century later, spoke even more strongly en the same lines. He said: "The law which inflicts penalty after penalty on a parent who is unwilling to have his child vaccinated is monstrous, and ought to be repealed." Admitting all that the most enthusiastic vaccinator claims for his operation, the writer cannot see why he, as a medical man, should make himself hated among his patients by striving to compel them to accept a benefit which they do not want. Vaccination we believe to be universally serviceable, and it is our duty to make it prevail universally by fair and reasonable tactics. But if, in spite of all this, people refuse to be vaccinated, they must be allowed to refuse; and they and their children must bear the consequences.

